# Supplementary material for: Identification of photoperiod-induced specific miRNAs in the adrenal glands of Sunite sheep (Ovis aries)
Source: Front Vet Sci. 2022 Jul 22;9:888207. doi: 10.3389/fvets.2022.888207 (PMC9354845; doi:10.3389/fvets.2022.888207)
Supplement: Supplementary file 1 [file Data_Sheet_1.ZIP › Supplementary file/miRDeep2 file.zip!/miRDeep2 file/Know_Structure/LP42A3/oar-mir-382.pdf]

miRBase precursor : oar-mir-382
Total read count : 6946
oar-miR-382-5p read count : 2581
oar-miR-382-3p read count : 4340
remaining reads : 25

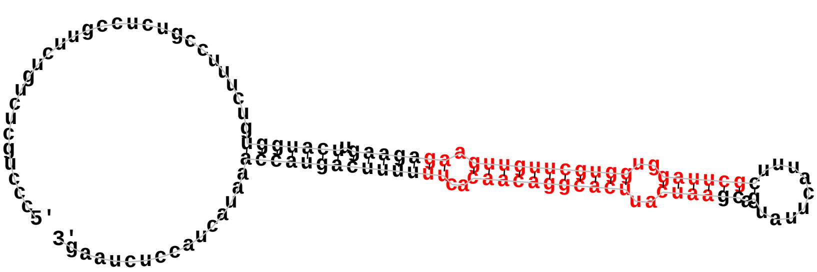

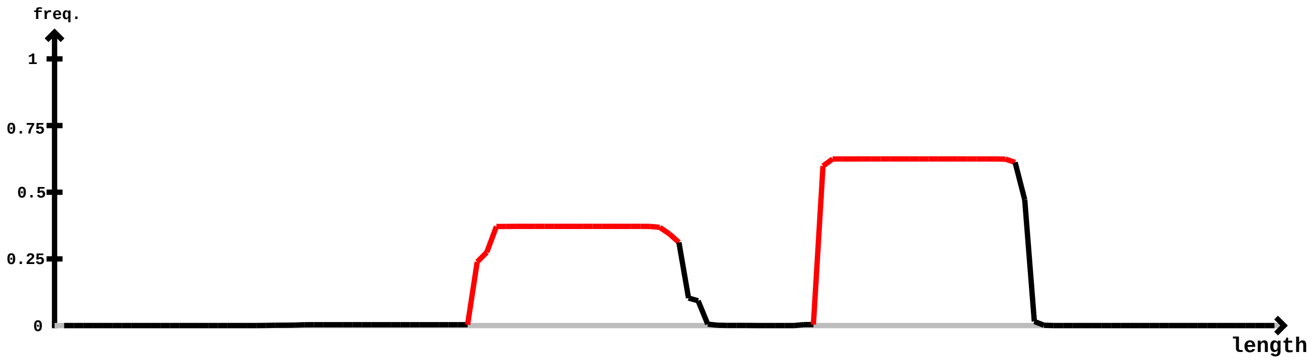

oar-miR-382-3p

oar-miR-382-5p

```
5'- cccugcucugucuugccucugccuuucugugguacuugaagagaaguuguucgugguggauucgcuuuacuuaugacgaaucauucacggacaacacuuuuuucaguaccaaauacuaccucuaag   -3'    exp
    ....((........)).............(((((((.(((((((.(((((((((((..(((((((.........).))))))..)))))))))))..))))))))))))))...............    reads     mm
    ....................gccuuucugugguacuugaaga....................................................................................    3         0
    .....................ccuuucugugguacuugaag.....................................................................................    1         0
    .....................ccuuucugugguacuugaaga....................................................................................    4         0
    .......................uuucugugguacuugaag.....................................................................................    1         0
    .......................uuucugugguacuugaaga....................................................................................    3         0
    ........................uucugugguacuugaaga....................................................................................    8         0
    ........................uucugugguacuugaagaga..................................................................................    1         0
    .........................ucugugguacuugaaga....................................................................................    1         0
    .........................................agaaguuguucgugguggauu................................................................    2         0
    ..........................................gaaguuguucguggugga..................................................................    2         0
    ..........................................gaaguuguucgugguggau.................................................................    24        0
    ..........................................gaaguuguucgugguggauu................................................................    158       0
    ..........................................gaaguuguucgugguggauuc...............................................................    208       0
    ..........................................gaaguuguucgugguggauucg..............................................................    1222      0
    ..........................................gaaguuguucgugguggauucgc.............................................................    39        0
    ..........................................gaaguuguucgugguggauucgcu............................................................    1         0
    ...........................................aaguuguucgugguggauu................................................................    7         0
    ...........................................aaguuguucgugguggauuc...............................................................    16        0
    ...........................................aaguuguucgugguggauucg..............................................................    220       0
    ...........................................aaguuguucgugguggauucgc.............................................................    9         0
    ...........................................aaguuguucgugguggauucgcu............................................................    1         0
    ............................................aguuguucgugguggauu................................................................    1         0
    ............................................aguuguucgugguggauucg..............................................................    7         0
    ............................................aguuguucgugguggauucgc.............................................................    24        0
    ............................................aguuguucgugguggauucgcu............................................................    606       0
    ............................................aguuguucgugguggauucgcuu...........................................................    28        0
    ............................................aguuguucgugguggauucgcuuu..........................................................    6         0
    ..............................................uuguucgugguggauucgcuuuacu.......................................................    3         0
    ............................................................................cgaaucauucacggacaac...............................    1         0
    ............................................................................cgaaucauucacggacaacac.............................    2         0
    ............................................................................cgaaucauucacggacaacacu............................    9         0
    ............................................................................cgaaucauucacggacaacacuu...........................    12        0
    .............................................................................gaaucauucacggacaacacuu...........................    2         0
    ..............................................................................aaucauucacggacaacac.............................    2         0
```

```
                                                                              oar-miR-382-3p
                                          oar-miR-382-5p
cccugcucugucuugccucugccuuucugugguacuugaagagaaguuguucgugguggauucgcuuuacuuaugacgaaucauucacggacaacacuuuuuucaguaccaaauacuaccucuaag

..............................................................................aaucauucacggacaacacu............................    75    0
..............................................................................aaucauucacggacaacacuu...........................   948    0
..............................................................................aaucauucacggacaacacuuu..........................  3034    0
..............................................................................aaucauucacggacaacacuuuu.........................    67    0
..............................................................................aaucauucacggacaacacuuuuu........................     3    0
...............................................................................aucauucacggacaacacuu...........................    11    0
...............................................................................aucauucacggacaacacuuu..........................   136    0
...............................................................................aucauucacggacaacacuuuu.........................    32    0
...............................................................................aucauucacggacaacacuuuuu........................     5    0
..................................................................................auucacggacaacacuuu..........................     1    0
```
